# Comorbidity of behavioral problems and parental acceptance-rejection in children diagnosed with chest discomfort, palpitations, vasovagal syncope, and underlying heart disease: a multiple case-control study

**DOI:** 10.1186/s12888-024-05527-3

**Published:** 2024-01-24

**Authors:** Yasemin Nuran Dönmez, Dilek Giray, Serdar Epcacan, Siddika Songül Yalçin

**Affiliations:** 1 Department of Pediatric Cardiology, Van Training and Research Hospital, Van, Turkey; 2 Department of Pediatrics, Faculty of Medicine, Hacettepe University, Ankara, Turkey

**Keywords:** Cardiac symptomatology, Pediatric cardiology outpatient clinic, Heart-related symptoms, Psychosocial factors, Behavioral problems

## Abstract

**Background:**

Children who experience chest discomfort, palpitations, vasovagal syncope, and underlying heart disease often present a complex clinical picture. Not only are they dealing with potential cardiac issues, but they may also exhibit behavioral problems that can complicate the diagnostic and treatment process. Moreover, parental acceptance or rejection can significantly influence the child’s well-being and medical outcomes in such cases. This study aims to explore the comorbidity of behavioral problems and parental acceptance-rejection in children diagnosed with these cardiac symptoms and underlying heart disease.

**Methods:**

In a case-control study, the Parental Acceptance - Rejection Questionnaire and Parental version of Strengths and Difficulties Questionnaire (SDQ) was filled by parents of 314 patients from pediatric cardiology clinic.

**Results:**

The control group scored substantially lower overall according to SDQ. The vasovagal syncope subgroup was found to have considerably lower scores on the subscale. The group with chest discomfort scored highly in hostility and aggression in the PARQ. In comparison to the other groups, the vasovagal syncope and chest pain group demonstrated higher scores in undifferentiated rejection and total score.

**Conclusion:**

This study showed a correlation between children’s behavioral and emotional problems and cardiac symptoms. This states that children’s relationship with their parents has an impact on their symptoms. It will be necessary to conduct further studies to determine a causal association and devise preventative measures.

**Supplementary Information:**

The online version contains supplementary material available at 10.1186/s12888-024-05527-3.

## Introduction

Children with congenital or acquired heart diseases may experience behavioral and emotional difficulties at a high rate, compromising their quality of life and straining their relationships with family members due to ongoing treatment procedures and hospital admissions [1, 2]. In the family of children with underlying heart disease, parental stress, despair, or anxiety can significantly influence a child’s upbringing. It is crucial to be aware of these factors as they can impact the child’s behavioral and psychosocial development, warranting attention [1, 3]. Additionally, pediatric cardiology clinics often encounter patients presenting with common symptoms like chest pain, palpitations, and fainting. Interestingly, most of these patients exhibit no discernible underlying heart disease [4, 5]. The high number of referrals to pediatric cardiology clinics, coupled with limited resources, results in financial and workload implications [4]. This, in turn, contributes to psychological distress in parent-child pairs, leading to increased anxiety levels for both children and their parents [6]. Frequent hospital admissions due to these symptoms may negatively impact both the parent’s quality of life and the child’s school attendance and academic performance [7]. Understanding and identifying the risk and environmental factors related to psychological aspects in these frequent referrals to the pediatric cardiology outpatient clinic helps in developing strategies to alleviate the cost burden, a critical step in effectively dealing with and achieving clinical success in practice. By evaluating family behaviors and the difficulties experienced by the child, it’s possible to prevent psychosocial factors from affecting mental health. This, in turn, helps establish healthy communication between the family and the child, ultimately reducing the severity of symptoms and hospital admissions [8]. Healthcare practitioners that are aware of psychosocial aspects are better able to provide patients and their parents with referrals to mental health professionals, address anxiety and behavioral issues, and improve quality of life using early cognitive therapies ease the financial strain on the healthcare system.

Children who perceive rejection from their parents often grow up to exhibit hostility, aggression, low self-confidence, emotional instability, and pessimism [9]. This is in line with attachment theory, which suggests that early experiences with caregivers, including parents, can significantly impact a child’s emotional and psychological development [10]. Insecure attachment patterns, characterized by parental rejection or neglect, can lead to the development of emotional and behavioral issues in children. Patients presenting with symptoms like chest pain, palpitations, and vasovagal syncope without underlying cardiac disease may strain the parent-child bond due to multiple hospital admissions, causing anxiety among families and children. Attachment theory also highlights the importance of a secure attachment between children and their caregivers for emotional well-being. Frequent hospitalizations and medical procedures can disrupt this attachment, leading to feelings of anxiety and insecurity in children [10]. Additionally, several variables, including family socioeconomic status, education level, family size, the number of family members, and genetic traits, can influence a child’s development and their relationship with their parents. Children deprived of parental attention may experience reduced functioning, difficulty managing stress, and psychological issues such as depression, anxiety, post-traumatic stress disorder, or behavioral problems, often leading to long-term health issues [11, 12]. This is consistent with the social learning theory, which posits that children learn behaviors and coping mechanisms through observation and interaction with their parents and caregivers [3]. Insufficient attention and negative family behaviors can contribute to the development of mental health issues in children. Family behaviors play a significant role in shaping children’s personalities. Negative traits like criticism, neglect, and hostility in the family environment can hinder a child’s development. Family systems theory underscores the interconnectedness of family members and how their interactions impact the family’s overall functioning [13]. Dysfunctional family behaviors can disrupt the family system and, consequently, affect the development of children. No prior studies have explored the connection between “dysfunctional family behavior and children’s problematic behavior” and the presence of chest discomfort, palpitations, or syncope in children. Our hypothesis is that symptoms like chest pain, palpitations, and vasovagal syncope, known to be influenced by psychosocial factors without underlying cardiac pathology, may be more prevalent in families exhibiting negative traits like aggression, neglect, or rejection.

The objective of this multiple case-control study is to investigate the status of behavioral problems and parental acceptance-rejection in groups of children presenting with specific cardiac symptoms including chest discomfort, palpitations, vasovagal syncope, and underlying heart disease. By analyzing this relationship, we seek to gain insights that can contribute to a better understanding of the challenges these children and their families face, ultimately improving the care and support provided to them in clinical settings.

## Materials and methods

### Settings

This multiple case-control study included children aged 9–18 years who applied to the Van Training and Research Hospital Pediatric Cardiology Outpatient Clinics in 2022. Van is the largest province in eastern Turkey, with a population of approximately 1.128 million, of which 32.5% are aged 0–14 [14].

The Ethics Board of Van Training and Research Hospital approved the protocol for this Non-Interventional Clinical Research (Issue Number: 2022/06 − 02). Children and their parents were informed about the study. Children provided verbal consent, while parents provided written consent to participate.

### Participants

Inclusion criteria: All parent-child pairs who met the inclusion criteria and voluntarily completed the forms during the study period were included in the study at the Pediatric Cardiology Outpatient Clinics.

Exclusion criteria: The study excluded children with illiterate parents, those with known psychiatric problems, children with mental and developmental delays, and children with chronic diseases other than heart disease. Additionally, children whose parents were separated or not living with them were also excluded from the study.

All children received a thorough cardiac examination to identify any underlying heart disease. Children diagnosed with an underlying cardiac disease were placed in the ‘having a cardiac disease’ group. Other children with a normal cardiac evaluation were assigned to the chest pain, vasovagal syncope, palpitation, and control groups based on their presenting cardiac symptomatology. Children without any complaints who applied for school sports group health reports were included in the control group.

### Variables

Socio-demographic characteristics, including the ages of the patients and their parents, the number of siblings, as well as the parents’ education levels and occupations, were documented on a study form. Subsequently, one parent individually completed both the Strengths and Difficulties Questionnaire (SDQ) and the standard version-parent form of Parental Acceptance-Rejection Questionnaire (PARQ) within 20 min.

### Instruments

**The strengths and difficulties questionnaire (SDQ)**, developed by Goodman, assesses the social, emotional, and behavioral functioning of patients [15]. It consists of 25 questions divided into 5 subgroups, including emotional symptoms, conduct problems, peer relationship problems, hyperactivity/inattention, and prosocial behavior. The sum of the first four subgroups yields the total difficulty score, with higher scores indicating more difficulty. To detect low and high probability psychiatric conditions, the SDQ is also evaluated using three components: internalizing, externalizing, and prosocial [16]. Scores from the subscales can be categorized to assess internalizing and externalizing problems. Externalizing problems encompass hyperactivity/inattention and conduct problems, while internalizing problems include emotional problems and peer issues. The Turkish version of the SDQ questionnaire’s validity and reliability were established by Guvenir et al. [17] with Cronbach’s alpha coefficient values of 0.22 to 0.84. In the present study, Turkish version of the SDQ questionnaire’s was used and Cronbach’s alpha coefficient values ranged from 0.50 to 0.83 in the SDQ (Table 1).

**Table 1:** Cronbach’s alpha values of subscales of Strengths and Difficulties Questionnaire and Parental Acceptance-Rejection/Control Questionnaire according to cardiac symptomatology, parents filling questionnaire, children’ age

	Item, no	Groups of cardiac symptomatology					Parents filling the Questionnaire		Children’ age, yr		
		Control	Palpitation	Chest Pain	Vasovagal Syncope	Having a cardiac disease	Mother	Father	< 12	≥ 12	Overall
*n*											316
**Strengths and Difficulties Questionnaire**											
Externalizing problems	10	0.70	0.65	0.70	0.78	0.70	0.70	0.72	0.69	0.72	0.71
Internalizing problems	10	0.50	0.61	0.59	0.65	0.60	0.57	0.64	0.68	0.55	0.60
Total problem score	20	0.70	0.73	0.71	0.83	0.78	0.73	0.79	0.76	0.76	0.76
Prosocial score	5	0.66	0.57	0.76	0.64	0.61	0.64	0.71	0.69	0.66	0.67
**Parental Acceptance-Rejection/Control Questionnaire**											
Warmth/Affection	20	0.74	0.80	0.72	0.94	0.80	0.81	0.85	0.77	0.84	0.82
Hostility/Aggression	15	0.88	0.77	0.83	0.88	0.76	0.84	0.83	0.73	0.86	0.83
Indifference/Neglect	15	0.75	0.66	0.79	0.83	0.67	0.77	0.73	0.71	0.77	0.75
Undifferentiated Rejection	10	0.58	0.61	0.74	0.81	0.62	0.74	0.69	0.49	0.77	0.71
Total PARQ score	60	0.91	0.90	0.91	0.94	0.91	0.91	0.92	0.87	0.93	0.92
Control	13	0.70	0.35	0.71	0.67	0.44	0.63	0.60	0.43	0.66	0.61

**Parental acceptance - rejection questionnaire (PARQ)** developed by Rohner, is used to explore the parent-child relationship and the impact of parental behavior on a child’s psychosocial development. It assesses two primary dimensions: ‘warmth’ and ‘behavioral control.’ The warmth dimension can be rated based on acceptance or rejection and includes four subscales: warmth/affection, hostility/aggression, neglect/indifference, and undifferentiated rejection. The ‘behavioral control’ dimension examines the level of constraint on the child’s behavior, distinguishing between permissiveness and strictness [18]. The scale comprises 73 questions with a 4-point Likert scale (1- never true, 2- rarely true, 3- sometimes true, 4- almost always true). A high score indicates a high level of rejection. PARQ measures the strength of the emotional connection between parents and children, as well as how parents express their feelings through actions. The Turkish version’s validity and reliability were assessed by Varan [19] for the PARQ/control questionnaire. In the present study, Cronbach’s alpha coefficients for the four PARQ scales ranged from 0.35 to 0.94, with the lowest values in the palpitation group’s control scale (alpha = 0.35) and the control group’s undifferentiated rejection scale (alpha = 0.58). In contrast, the vasovagal syncope group showed Cronbach’s alpha coefficients greater than 0.80 for all PARQ subscales.

### Study sample size

Total sample size was calculated as 303 and determined by effect size = 0.25 (medium), alpha error = 0.05, power = 0.95, number of groups = 5 with ANCOVA: fixed effects (G*Power 3.1.9.4).

### Statistical methods

The data was analyzed by IBM-SPSS statistics version 22.0. Cronbach’s alpha values of SDQ and PARQ scores were calculated. Distribution characteristics of variables were evaluated with Kolmogorov Smirnov test and histogram. Mean with standard deviation, median with 25–75 percentile values and frequency with percentiles were calculated. According to variable characteristics, groups were compared with One-way Anova with Duncan test for subgroup analysis or Kruskal-wallis Anova test with pairwise comparison. For categorical variables, differences among groups were analyzed with the chi-square test, and groups that made differences were identified with residual analysis and Bonferroni correction.

Generalized Linear Models analysed the association between groups and each subscore (both SDQ and PARQ) after controlling for age groups (yrs), child’s gender, parental age (yrs) and child number. When a significant association was detected in groups, pairwise contrast was performed with least significant difference (LSD). Estimated means with standart errors was given.

*P* value less than 0.05 was taken as significant.

## Results

A total of 316 children and their parents were included in the study (Supplementary Table 1). Patients were grouped based on their reasons for admission to pediatric cardiology outpatient clinics. These groups consisted of 73 patients with palpitations, 70 with non-cardiac chest pain, 55 with vasovagal syncope, 61 with underlying heart conditions, and 57 who served as the control group. The cardiac group consisted of septal defects (atrial septal defect, ventricular septal defect, atrioventricular septal defect), right-sided outflow tract obstruction (pulmonary atresia with intact ventricular septum and pulmonary stenosis), left-sided outflow obstruction (coarctation of the aorta), and other cardiac structural abnormalities (atrioventricular valve regurgitation, bicuspid aortic valve, semilunar valve regurgitation and rheumatic heart disease).

Nearly half of the forms was filled by mother (52.8%).

Family characteristics were presented in Table 2. In this regard, no significant differences were observed among the groups regarding the age and gender of the enrolled children, maternal and paternal age, maternal education level, and maternal employment status. There was a statistically significant difference in father education level was found between the control group and the other groups (*p* < 0.05).

**Table 2:** Baseline characteristics, *n* = 316

	Control (*n* = 57)	Palpitation (*n* = 73)	Chest Pain (*n* = 70)	Vasovagal Syncope (*n* = 55)	Underlying heart conditions (*n* = 61)	*p*
Child’s age, yrs	12.8 ± 2.4	12.6 ± 3.1	12.4 ± 3.0	12.8 ± 2.7	13.1 ± 2.8	0.741*
Child’s age < 12 yrs	24.6	38.4	40.0	29.1	24.6	0.157**
Sex, male	57.9	45.2	40.0	41.8	49.2	0.301**
Child number	3.0 ± 1.0	3.5 ± 1.3	3.4 ± 1.4	3.4 ± 1.4	3.7 ± 1.5	0.087*
Parent filling out the form, mother	40.4	49.3	58.6	60.0	55.7	0.185
Mother age, yrs	37.7 ± 4.4	38.7 ± 7.0	40.0 ± 5.6	39.2 ± 5.7	39.8 ± 4.2	0.142*
Father age, yrs	41.2 ± 4.8	42.5 ± 6.7	43.2 ± 4.9	43.1 ± 5.6	44.2 ± 4.9	0.056*
Age of parent filling out the form	39.8 ± 4.6	40.3 ± 6.8	41.3 ± 5.7	40.5 ± 6.0	41.7 ± 4.8	0.315*
Mother’s education ≥ high school	28.9	17.8	11.4	12.7	14.8	0.058**
Father’s education ≥ high school	52.6^a^	27.4^b^	30.0^b^	25.5^b^	27.9^b^	0.009**
Education of parent filling out the form ≥high school	43.9^a^	24.7^b^	20.0^b^	20.0^b^	21.3^b^	0.013**
Mother’s having a job	12.3	16.4	5.7	7.3	8.2	0.221**

Values were mean ± SD or %

*One-way Anova

**Chi-square test, groups that made differences were identified with residual analysis and Bonferroni correction; ^ab^Different letter in the same row showed significant differences between values (*p* < 0.05)

The control group exhibited significantly lower median scores for hyperactivity problems and emotional problems compared to other groups with cardiac symptomatology (*p* = 0.031 and *p* = 0.001, respectively; Table 3). The median scores for internalizing problems and the total SDQ were also lower in the control group when compared to the groups experiencing palpitation and vasovagal syncope (*p* = 0.012 and *p* = 0.005, respectively).

**Table 3:** The median levels of Strengths and Difficulties Questionnaire and Parental Acceptance-Rejection/Control Questionnaire scores among groups

	Control (*n* = 57)	Palpitation (*n* = 73)	Chest Pain (*n* = 70)	Vasovagal Syncope (*n* = 55)	Underlying heart conditions (*n* = 61)	*p*
**Strengths and Difficulties Questionnaire**						
Conduct problems	2 (1–4)	3 (1–4)	2 (1–4)	3 (1–5)	2 (1–4)	0.210
Hyperactivity problems	3 (2–5)^a^	4 (3–6)^b^	4 (2–6)^b^	4 (3–6)^b^	4 (3–6)^b^	0.031
Emotional problems	2 (1–4)^a^	4 (2–6)^b^	3 (2–6)^b^	4 (2–5)^b^	3 (2–6)^b^	0.001
Peer-problems	3 (2–4)	3 (2–4)	3 (2–4)	3 (2–5)	3 (2–4)	0.470
Internalizing problems	5 (2–7)^a^	7 (5–9)^b^	7 (4–9)	7 (4–10)^b^	6 (4–10)	0.012
Externalizing problems	4 (3–9)	7 (5–9)	7 (4–9)	7 (4–11)	7 (4–10)	0.067
Total score	10 (6–15)^a^	15 (9–18)^b^	13 (9–17)	13 (9–21)^b^	12 (9–19)	0.005
Prosocial score	10 (8–10)^a^	9 (8–10)	9 (7–10)	8 (7–10)^b^	8 (7–10)^b^	0.007
**Parental Acceptance-Rejection/Control Questionnaire**						
Warmth/Affection	26 (23–28)	27 (23–32)	27 (24–32)	28 (23–33)	27 (23–33)	0.576
Hostility/Aggression	19 (17–25)	22 (18–27)	24 (18–28)	22 (18–28)	20 (18–24)	0.068
Indifference/Neglect	19 (16–24)	21 (17–24)	20 (17–25)	21 (18–27)	19 (16–23)	0.200
Undifferentiated Rejection	12 (10–15)^a^	14 (12–16)	14 (12–18)^b^	13 (12–17)	13 (11–14)	0.003
Total	76 (69–93)	83 (73–95)	85 (73–100)	85 (76–97)	80 (71–92)	0.064
Control subscale scores	40 (36–44)	41 (38–43)	42 (40–45)	40 (35–44)	41 (38–44)	0.064
Strictness control, %	52.6^a^	67.1^ab^	78.6^b^	54.5^a^	62.3^a^	0.016

Values median (25–75 percentile) or %

Kruskal Wallis test; ^ab^Different letter in the same row showed significant differences between values (*p* < 0.05)

However, the control group showed higher prosocial scores than cases having vasovagal syncope and underlying heart conditions. Regarding the PARQ/C median undifferentiated rejection scores, they were lower in the control group in comparison to the cases having chest pain (*p* = 0.003). While there were no statistically significant differences among the groups in the median control score, it is worth noting that the strictness in the control scale of PARQ/C were detected to be the highest in cases with chest pain. In addition, the strictness control score was also observed to be lower in the vasovagal syncope and control groups.

After adjusting for age groups, gender, parental age and the number of children, generalized linear models revealed differences in hyperactivity problems, emotional problems, internalizing problems, total SDQ score and prosocial score among groups (Table 4). The chest pain group exhibited the highest scores for hostility and aggression subscales. The vasovagal syncope and chest pain group demonstrated higher scores in undifferentiated rejection and total score compared to the other groups. Total PARQ scores were observed to be lowest in control and cases having underlying heart conditions. No significant differences were observed in warmth/affection and indifference/neglect between the groups.

**Table 4:** Differences in Strengths and Difficulties Questionnaire and Parental Acceptance-Rejection/Control Questionnaire scores among groups, Generalized Linear Models

	Control (*n* = 57)	Palpitation (*n* = 73)	Chest Pain (*n* = 70)	Vasovagal Syncope (*n* = 55)	Underlying heart conditions (*n* = 61)	
						*p*
**Strengths and Difficulties Questionnaire**						
Conduct problems	2.30 ± 0.40	2.80 ± 0.34	2.70 ± 0.37	3.11 ± 0.40	2.27 ± 0.38	0.184
Hyperactivity problems	3.18 ± 0.41^a^	4.16 ± 0.34^b^	4.16 ± 0.38^b^	4.24 ± 0.41^b^	4.42 ± 0.38^b^	0.032
Emotional problems	2.54 ± 0.45^a^	4.15 ± 0.37^b^	3.87 ± 0.41^b^	4.18 ± 0.44^b^	3.98 ± 0.42^b^	0.001
Peer-problems	3.13 ± 0.30	2.91 ± 0.25	3.03 ± 0.28	3.45 ± 0.30	3.15 ± 0.28	0.438
Internalizing problems	5.66 ± 0.62^a^	7.06 ± 0.52^b^	6.91 ± 0.58^b^	7.63 ± 0.62^b^	7.13 ± 0.58^b^	0.034
Externalizing problems	5.48 ± 0.70	6.96 ± 0.59	6.85 ± 0.65	7.35 ± 0.70	6.69 ± 0.66	0.105
Total score	11.14 ± 1.12^a^	14.03 ± 0.94^b^	13.76 ± 1.04^b^	14.98 ± 1.12^b^	13.82 ± 1.05^b^	0.016
Prosocial score	8.92 ± 0.34^a^	8.60 ± 0.28^ab^	8.20 ± 0.31^bc^	7.93 ± 0.33^c^	8.17 ± 0.31^bc^	0.031
**Parental Acceptance-Rejection/Control Questionnaire**						
Warmth/Affection	25.9 ± 1.5	27.2 ± 1.3	27.8 ± 1.4	29.2 ± 1.5	26.8 ± 1.4	0.271
Hostility/Aggression	22.3 ± 1.3^ab^	24.1 ± 1.1^bc^	25.3 ± 1.2^c^	24.8 ± 1.3^bc^	22.0 ± 1.2^a^	0.027
Indifference/Neglect	20.9 ± 1.0	21.1 ± 0.9	22.0 ± 1.0	23.0 ± 1.4	20.2 ± 1.0	0.055
Undifferentiated Rejection	13.2 ± 0.8^a^	14.8 ± 0.6^bc^	16.0 ± 0.7^c^	15.4 ± 0.8^c^	13.4 ± 0.7^ab^	< 0.001
Total score	82.4 ± 3.7^a^	86.8 ± 3.2^ab^	90.8 ± 3.5^b^	92.3 ± 3.7^b^	82.8 ± 3.5^a^	0.020
Control subscale scores	40.2 ± 0.9	40.3 ± 0.8	41.5 ± 0.8	39.4 ± 0.9	40.4 ± 0.8	0.169

*Estimated Mean ± SEM, Generalized Linear Models adjusted for age groups, gender, parental age and child number, and pairwise contrast performed with LSD

^abc^Different letter in the same row showed significant differences between values (*p* < 0.05)

## Discussion

The present study found a connection between children’s behavioral and emotional issues and their cardiac symptoms. Furthermore, our research indicated that children’s symptoms were influenced by their family acceptance-rejection.

Our study revealed a relationship between a decrease in the father’s education level and cardiac symptoms in children, such as palpitations, vasovagal syncope, or chest pain. The level of family education is known to be linked to a child’s mental health [20, 21]. This might be due to the stress factors that arise in children when fathers have lower education levels, resulting in psychosomatic symptoms and a strained father-child bond. Family characteristics, such as parental occupation, education level, and time spent away from the child, play a significant role in this relationship [22]. Children who have close, supportive relationships with their parents tend to cope better with life’s challenges and experience fewer mental health problems.

In our study, we aimed to investigate whether a child’s cardiac symptoms were associated with parental acceptance or rejection. According to our findings, children of aggressive parents frequently reported chest pain. Vasovagal syncope and chest pain were also more common in children whose parents did not pay attention to them. This suggests that children experiencing vasovagal syncope or chest pain may not be receiving sufficient attention, which could lead to psychological factors related to a need for affection. Low attention and excessive parental protection have previously been associated with chronic pain [23], and our results suggest a link between low care and vasovagal syncope alongside ongoing pain.

Children with underlying cardiac disease and those in the control group with good health had lower total PARQ scores and rejection scores (aggression, neglect, undifferentiated rejection). This indicates that families with children who have heart problems tend to have better relationships and display more compassion for their child. This finding aligns with previous research indicating that children with congenital heart disease experience less depression when their parents are supportive and encouraging [24]. Effective communication and encouragement from parents have been associated with lower depression rates and higher quality of life for children [25]. Families of children with underlying heart conditions may exhibit more protective behavior due to fear of losing their child [26], which could explain the lower rejection scores. However, our study did not find a difference in behavioral control, suggesting that families were not overly protective or controlling toward their children. Further research is needed to understand how children perceive these findings. The frequency, severity, and duration of a child’s exposure to rejection can impact their psychological, developmental, and physical health [12]. Sensitive individuals may experience social pain related to rejection in a way similar to physical pain, which can decrease when social discomfort is minimized [27]. Therefore, reducing the perception of rejection and maintaining better family-child relationships may lead to a decrease in physical effects. Some studies have suggested a link between childhood emotional neglect and later health issues, such as cerebral infarction [28]. Further research is required to explore the potential connection between parental rejection and negative cardiovascular effects.

Our findings from parental SDQ assessments revealed that the total difficulty scores of children and adolescents with cardiac symptoms and underlying heart problems were significantly higher compared to the healthy group. Additionally, we observed lower prosocial scores in the vasovagal syncope group. This suggests that children in this group may exhibit fewer positive social behaviors compared to other groups. These lower prosocial scores could indicate potential challenges in interpersonal relationships among children with syncope symptoms.In a study by Bolat et al., adolescents with non-cardiac chest pain were more likely to exhibit conduct problems [29]. However, in our analysis, there were no statistically significant associations between chest pain and conduct problems. Children with somatization complaints tend to use fewer coping strategies and may exhibit internalizing and insecure characteristics. They often express their emotional problems through physical symptoms [8]. Therefore, conducting family-focused psychological assessments is essential. Psychological factors, especially maternal depression, can contribute to children with chest pain seeking medical attention, as it has been observed that maternal depression increases the likelihood of children experiencing chest pain [30]. Yoldas et al. [31] reported that the non-cardiac chest pain group in a preschool setting had higher levels of behavioral problems, particularly internalized problems, and anxiety. They also found that lower maternal education levels were associated with higher scores in emotionally reactivated, anxiety/somatic complaints, and internalized problems [31]. According to Lipsitz’ study, anxiety disorders such as panic disorder, social phobia, and generalized anxiety disorder may be common in children with non-cardiac chest pain [32]. Children with non-cardiac chest discomfort are also known to experience significant levels of anxiety and anxiety sensitivity [33]. Syncope and somatic symptoms have been linked to both parents’ and children’s psychological well-being in previous studies [34, 35]. Even in children without underlying structural cardiac diseases, no history of arrhythmia, and normal test results, psychological issues such as despair, panic disorders, and anxiety can still manifest [36, 37]. It is evident that the relationships formed between parents and children during childhood, along with the attitudes and behaviors of parents towards their children, significantly influence the child’s psychological, social, and emotional development. These factors may also have a connection to cardiac symptomatology.

### Strengths and limitations

In this study, both SDQ and PARQ were performed in children with non-cardiac chest pain, palpitation, vasovagal syncope, and structural heart disease who applied to the pediatric cardiology outpatient clinic. To the best of our knowledge, this is the first study to investigate the relationship between them.

While the sample sizes for each group were small, the results are promising in terms of shedding light on the connection between children and their families and addressing potential issues. As with previous studies, our results also indicated low Cronbach alpha values for the SDQ. This suggests that there is a need for further research and development of new assessment scales.

Unfortunately, we were unable to assess the children’s perceptions as we did not want them to fill out the questionaries. Therefore, we couldn’t make direct comparisons regarding how the children evaluated their family’s behavior.

Our study yielded unique results specific to the cultural and social context of the region where the research was conducted. This region is more rural and characterized by traditional family structures compared to urban areas. Therefore, conducting a large-scale and multicentric study in various settings is essential for comprehensive insights. Additionally, future research should consider implementing self-report scales for children to gather their perspectives.

## Conclusion

In summary, this study has revealed a significant association between child problem behaviors and parental acceptance-rejection in relation to the child’s presenting complaint, which includes chest pain, palpitations, and vasovagal syncope. The PARQ can serve as a valuable tool in the field of pediatric cardiology to assess the psychosocial impact of parental acceptance or rejection during childhood. It can aid in investigating the underlying causes of cardiac symptomatology and provide insights into potential interventions. Understanding the factors contributing to behavioral health problems and their influence on parent-child interactions is crucial for addressing these issues effectively. Further research is necessary to establish causal relationships and develop preventive measures. Clinicians should be aware of the importance of parent-child relationships and how disruptions in this relationship may contribute to the development of medical conditions such as cardiac symptoms and vasovagal syncope.

### Electronic supplementary material

Below is the link to the electronic supplementary material.

Supplementary Material 1: Supplementary Table 1. General characteristic of enrolled children

## Data Availability

The datasets used and/or analysed during the current study are available from the corresponding author on reasonable request.
