# Supplementary material for: Comorbidity of behavioral problems and parental acceptance-rejection in children diagnosed with chest discomfort, palpitations, vasovagal syncope, and underlying heart disease: a multiple case-control study
Source: BMC Psychiatry. 2024 Jan 24;24:70. doi: 10.1186/s12888-024-05527-3 (PMC10809743; doi:10.1186/s12888-024-05527-3)
Supplement: Supplementary file 1 — Supplementary Material 1: Supplementary Table 1. General characteristic of enrolled children [file 12888_2024_5527_MOESM1_ESM.docx]

Supplementary Table 1. General characteristic of enrolled children

| Variable |  | Questionaires |  |
| --- | --- | --- | --- |
| Child’s age, yrs | 12.7±2.8 | **SDQ** |  |
| Child’s age <12 yrs | 32.0 | Conduct problems | 2 (1-4) |
| Sex, male, % | 46.5 | Hyperactivity problems | 4 (2-6) |
| Total number of children | 3.4±1.3 | Emotional problems | 3 (1-5) |
| 1-2 children | 26.6 | Peer-problems | 3 (2-4) |
| 3-4 children | 57.3 | Internalizing problems | 6 (4-9) |
| ≥5 children | 16.1 | Externalizing problems | 7 (4-10) |
| Parent filling out the form, mother, % | 52.8 | Total SDQ score | 13 (9-18) |
| Mother age, yrs | 39.1±5.6 | Prosocial score | 9 (7-10) |
| ≥40yrs | 44.6 |  |  |
| Father age, yrs | 42.8±5.5 | **PARQ/C** |  |
| ≥40yrs | 69.0 | Warmth/Affection | 27 (23-32) |
| Age of parent filling out the form | 40.7±5.7 | Hostility/Aggression | 21 (18-27) |
| ≥40yrs, % | 57.0 | Indifference/Neglect | 20 (17-24) |
| Mother’s education ≥high school | 17.1 | Undifferentiated Rejection | 13 (12-16) |
| Father’s education ≥high school | 32.3 | TOTAL PARQ | 82 (72-96) |
| Education of parent filling out the form≥high school, % | 25.6 | Control subscale scores | 41 (38-44) |
| Mother’s having a job, % | 10.1 | Control groups, % |  |
| Child groups, % |  | Low (13-26) | 0.9 |
| Control | 18.0 | Middle (27-39) | 35.1 |
| Palpitation | 23.1 | Strict (40-45) | 49.1 |
| Chest Pain | 22.2 | Restrictive (46-52) | 14.9 |
| Syncope | 17.4 |  |  |
| Underlying heart conditions | 19.3 |  |  |

Values were mean±SD, median (25-75 percentile) or %.
